# CHALLENGES AND REWARDS EXPERIENCED IN THE VERY OLD PARENT-CHILD RELATIONSHIP: RELATIONSHIP QUALITY MATTERS

**DOI:** 10.1093/geroni/igac059.2652

**Published:** 2022-12-20

**Authors:** Kathrin Boerner, Kyungmin Kim, Yijung Kim, Elizabeth Gallagher, Daniela Jopp

**Affiliations:** University of Massachusetts Boston, Boston, Massachusetts, United States; Seoul National University, Seoul, Seoul-t'ukpyolsi, Republic of Korea; The University of Texas at Austin, Austin, Texas, United States; University of Massachusetts Boston, Boston, Massachusetts, United States; University of Lausanne, Lausanne, Vaud, Switzerland

## Abstract

Very old parents and their “old” children are a growing group in industrialized countries worldwide. However, little is known about the nature and implications of this relationship constellation, especially the challenges and/or rewards experienced within the relationship. We therefore examined factors associated with perceptions of challenge and reward among very old parents and their children. Using data from 114 very old parent-child dyads in the Boston Aging Together Study, we estimated Actor-Partner Interdependence Models to predict challenge, reward, and challenge/reward ratio outcomes of dyad members as a function of relationship quality, support exchanges, family norms, and personality. Relationship quality emerged as the most influential predictor, albeit more consistently for children than for parents. When children experienced the parent-child relationship as more positive, both children and parents experienced fewer challenges and more rewards (i.e., both actor and partner effect). Parents’ experience of relationship quality was only associated with their own challenge perceptions (i.e., actor effect only). The roles of support exchanges, family norms, and personality were relatively minor, with few significant effects if at all. Given the importance of relationship quality for challenge and reward perceptions, support services or interventions targeting relationship quality could be a key pathway to minimizing challenges and maximizing rewards among very old parents and their children. Focusing on relationship perceptions of the child may be particularly critical in improving the experiences of both parent and child.

